# Supplementary material for: Comparative genomics provides new insights into the diversity, physiology, and sexuality of the only industrially exploited tremellomycete: Phaffia rhodozyma
Source: BMC Genomics. 2016 Nov 9;17:901. doi: 10.1186/s12864-016-3244-7 (PMC5103461; doi:10.1186/s12864-016-3244-7)
Supplement: Additional file 6: — List of orphan genes with links to PFAM (related to Additional file 1: Table S1). (ZIP 1428 kb) [file 12864_2016_3244_MOESM6_ESM.zip › BLAST_HTML_FTR/G04559_P.html]

BLAST Search Results


```
BLASTP 2.2.27+


Reference:
Stephen F. Altschul, Thomas L. Madden, Alejandro A. Schäffer,
Jinghui Zhang, Zheng Zhang, Webb Miller, and David J. Lipman (1997),
"Gapped BLAST and PSI-BLAST: a new generation of protein database
search programs", Nucleic Acids Res. 25:3389-3402.


Reference for
composition-based statistics:
Alejandro A. Schäffer, L. Aravind, Thomas L. Madden, Sergei
Shavirin, John L. Spouge, Yuri I. Wolf, Eugene V. Koonin, and
Stephen F. Altschul (2001), "Improving the accuracy of PSI-BLAST
protein database searches with composition-based statistics and
other refinements", Nucleic Acids Res. 29:2994-3005.


Database: nr
           71,551,133 sequences; 26,053,659,533 total letters


Query= G04559_P

Length=440
                                                                      Score     E
Sequences producing significant alignments:                          (Bits)  Value

emb|CED83322.1|  hypothetical protein [Xanthophyllomyces dendrorh...   885    0.0  


 >emb|CED83322.1| hypothetical protein [Xanthophyllomyces dendrorhous]
Length=667

 Score =  885 bits (2286),  Expect = 0.0, Method: Compositional matrix adjust.
 Identities = 439/439 (100%), Positives = 439/439 (100%), Gaps = 0/439 (0%)

Query  1    MATVFVNTQGTLNTPPDSHSTKTSTAESECDHAAGWSSSDVQLDSHPSASSSVPIPAVSS  60
            MATVFVNTQGTLNTPPDSHSTKTSTAESECDHAAGWSSSDVQLDSHPSASSSVPIPAVSS
Sbjct  229  MATVFVNTQGTLNTPPDSHSTKTSTAESECDHAAGWSSSDVQLDSHPSASSSVPIPAVSS  288

Query  61   GWVMKESATQQPQPTTTVPEEGIPSKVFHPGSRVGQESTFLNQMFPNHIPFNPQGSSHLS  120
            GWVMKESATQQPQPTTTVPEEGIPSKVFHPGSRVGQESTFLNQMFPNHIPFNPQGSSHLS
Sbjct  289  GWVMKESATQQPQPTTTVPEEGIPSKVFHPGSRVGQESTFLNQMFPNHIPFNPQGSSHLS  348

Query  121  SHSLYNSAPYHYPYNPYQAQPPFYSQAESYPNVFATGRPSVPTHLFPPHLRPQSEKLAEG  180
            SHSLYNSAPYHYPYNPYQAQPPFYSQAESYPNVFATGRPSVPTHLFPPHLRPQSEKLAEG
Sbjct  349  SHSLYNSAPYHYPYNPYQAQPPFYSQAESYPNVFATGRPSVPTHLFPPHLRPQSEKLAEG  408

Query  181  QESSNVASAPMTKRNSDGWLPYSPARTSISRASVMSDPTGRSSPNVSSLSPRHTPLVPLP  240
            QESSNVASAPMTKRNSDGWLPYSPARTSISRASVMSDPTGRSSPNVSSLSPRHTPLVPLP
Sbjct  409  QESSNVASAPMTKRNSDGWLPYSPARTSISRASVMSDPTGRSSPNVSSLSPRHTPLVPLP  468

Query  241  PPSADSVNDKLTVQVTSPTLSDTQSMSKARLTSLPHLQTSFLGAPPPLIPANNADPDRSQ  300
            PPSADSVNDKLTVQVTSPTLSDTQSMSKARLTSLPHLQTSFLGAPPPLIPANNADPDRSQ
Sbjct  469  PPSADSVNDKLTVQVTSPTLSDTQSMSKARLTSLPHLQTSFLGAPPPLIPANNADPDRSQ  528

Query  301  TEHASLRSQTNDATSLHIPGPSPISAYGHSISGGLWGGPPTGFPPFTPSGGMSSMYPISA  360
            TEHASLRSQTNDATSLHIPGPSPISAYGHSISGGLWGGPPTGFPPFTPSGGMSSMYPISA
Sbjct  529  TEHASLRSQTNDATSLHIPGPSPISAYGHSISGGLWGGPPTGFPPFTPSGGMSSMYPISA  588

Query  361  GSGATLGQGLGSGTGTGPWPWSLSGLGLSPKGAEREGEWEFGLMEEKTPMGGLTSSMEHR  420
            GSGATLGQGLGSGTGTGPWPWSLSGLGLSPKGAEREGEWEFGLMEEKTPMGGLTSSMEHR
Sbjct  589  GSGATLGQGLGSGTGTGPWPWSLSGLGLSPKGAEREGEWEFGLMEEKTPMGGLTSSMEHR  648

Query  421  IKGSPEKTQPPKTDLPDLS  439
            IKGSPEKTQPPKTDLPDLS
Sbjct  649  IKGSPEKTQPPKTDLPDLS  667


Lambda      K        H        a         alpha
   0.309    0.127    0.387    0.792     4.96 

Gapped
Lambda      K        H        a         alpha    sigma
   0.267   0.0410    0.140     1.90     42.6     43.6 

Effective search space used: 4299948524586


  Database: nr
    Posted date:  Sep 23, 2015 12:05 AM
  Number of letters in database: 26,053,659,533
  Number of sequences in database:  71,551,133


Matrix: BLOSUM62
Gap Penalties: Existence: 11, Extension: 1
Neighboring words threshold: 11
Window for multiple hits: 40
```
